# Exploring occupational therapy interventions in homeless settings in Tshwane

**DOI:** 10.4102/hsag.v31i0.3140

**Published:** 2026-03-05

**Authors:** Sarah Ainsworth, Helga E. Lister, Michelle N.S. Janse van Rensburg

**Affiliations:** 1 Department of Occupational Therapy, Faculty of Health Sciences, University of Pretoria, Pretoria, South Africa; 2 Department of Occupational Therapy, Faculty of Health Sciences, University of the Witwatersrand, Johannesburg, South Africa; 3 Community Oriented Primary Care (COPC) Research Unit, Department of Family Medicine, Faculty of Health Sciences, University of Pretoria, Pretoria, South Africa; 4 Unit for Street Homelessness, Faculty of Theology and Religion, University of Pretoria, Pretoria, South Africa

**Keywords:** occupational therapy, homelessness, Tshwane, Capability Approach, homeless settings

## Abstract

**Background:**

In South Africa, the national lockdown was both a challenge and an opportunity to address health-related issues of homelessness. While initial emergency responses in Tshwane focused on basic needs and medical intervention, the need for more holistic intervention became evident.

**Aim:**

To understand the experiences and perceptions, benefits and facilitators, and barriers and challenges of university-driven occupational therapy interventions with people experiencing homelessness in Tshwane during the various coronavirus disease 2019 lockdown levels and beyond to March 2023.

**Setting:**

Research was conducted in various homeless settings, such as temporary shelters, transitional- or permanent housing facilities or drop-in centres, where occupational therapy intervention was implemented.

**Methods:**

An explorative, descriptive qualitative design was used, with focus group discussions and semi-structured interviews conducted with people experiencing homelessness and homeless practitioners. These were audio recorded, transcribed and thematically analysed. Student-written occupational therapy planning and feedback documents were analysed through reflexive thematic analysis and interpreted using the Capability Approach.

**Results:**

Inputs and conversion factors positively influencing intervention outcomes included a supportive environment, client-centred care, therapeutic activities in small groups, ensuring positive volition, and understanding the real-life experiences of participants. Challenges included unsupportive environments, language barriers, poor attitudes and motivation, and lack of continuity of occupational therapy intervention.

**Conclusion:**

Occupational therapy intervention in homeless settings is valuable, as it considers personal, social and environmental aspects affecting a person’s functioning.

**Contribution:**

Applying the unique contribution of occupational therapy intervention in homeless settings will improve overall health- and social service delivery.

## Introduction

Homelessness is a global phenomenon, with an estimated 100 million homeless people worldwide, and 1.6 billion people lacking adequate housing (Homeless World Cup Foundation 2024). There are varying definitions of homelessness across countries, creating different perceptions and policy priorities (Salcedo 2019). Simply put, homelessness can be understood as individuals who have no home. However, the extent to which people experiencing homelessness are affected goes beyond the lack of a physical shelter, because homelessness is both a result of and a contributing factor to the denial, abuse and violation of human rights (Salcedo 2019).

Causes of homelessness include structural factors such as housing shortages, limited availability of affordable housing, high unemployment rates, job losses and rapid urbanisation (Cuthill 2019; Khadduri & Shinn 2020). Personal or individual factors related to homelessness can include domestic abuse; mental health issues; drug, alcohol and gambling addiction; low levels of education; long stay in a correctional facility; life crises; bereavement; relationship breakdowns; and lack of support (Mbewu & Tenai 2020). Pathways into homelessness may vary depending on an individual’s life stage. For young people this may include family instability and conflict, a history of out-of-family-care, a young person’s ‘problem behaviour’ or negative peer relationships, whereas for older people, this may include relationship losses, widowhood or retirement, coupled with personal vulnerability associated with ageing (Joly, Cornes & Manthorpe 2014).

People experiencing homelessness are faced with numerous challenges. Homelessness negatively impacts one’s physical and mental well-being, and those experiencing homelessness are considered vulnerable to severe health conditions and increased morbidity when compared to the general public (Pottie 2020). They experience social stigma and marginalisation, are at risk of developing serious mental health issues (depression, anxiety, substance use), are at higher risk of medical comorbidities (Salisbury-Afshar, Rich & Adashi 2020) or other diseases and chronic conditions (HIV, tuberculosis, diabetes, cancer, respiratory problems, heart disease), have difficulties accessing health care because of stigma, and experience reduced safety and security, and are, thus, at risk of further violence (assault, rape, abuse) (Davies & Wood 2018; Scaffa 2014). Furthermore, people experiencing homelessness face barriers in applying for benefits such as social grants because of missing identity and other essential documents, as well as difficulty obtaining these documents (Cross et al. 2010). Other barriers may include limited transportation to attend job interviews or classes, poor literacy skills and insufficient resources, which prevent access to health and social services (Roy et al. 2017; Schultz-Krohn & Tyminski 2018).

Homelessness in South Africa is a dynamic issue requiring multi-sectoral involvement (Roets et al. 2016). The drivers of homelessness in South Africa include historical disadvantage, migration, unemployment and low wages, social exclusion, loss of parents or a household breadwinner, home desertion and missing identification documents (Obioha 2021). Homelessness is addressed through the Bill of Rights, which states that all citizens should have access to sufficient housing, healthcare, basic sanitation and hygiene, social security and assistance, as well as the right to receive emergency medical treatment without refusal. South African legislation such as *The Social Assistance Act, the National Health Act, the Housing Act* and the *Children’s Act* (Naidoo 2010) also speak to issues of homelessness.

The 2022 South African National Census (Statistics South Africa 2023) estimated 55 719 homeless people in the country, with the City of Tshwane Metropolitan Municipality (CoTMM) forming the largest proportion of South Africa’s homelessness population at 18.1%. These figures are currently being debated, however, as there appears to be an overestimation in some settings and an underestimation in others (Statistics South Africa 2023). A study conducted in October 2022, ‘Everyone counted, counts’, was the first homeless count conducted across the CoTMM seven regions and determined the number of people experiencing homelessness (i.e. people ‘sleeping under the stars’) in Tshwane to be around 5000 (De Beer 2022). Either way, there is an increase in street homelessness, with an upward trajectory for which South Africa is not prepared (Mbewu & Tenai 2020).

### Background

The novel coronavirus, commonly known as COVID-19, infected people across the globe, and was declared a pandemic by the World Health Organization on 11 March 2020 (Parkes et al. 2021). In South Africa, the emerging pandemic was recognised by the Government as a risk, and the country declared a National State of Disaster on 15 March 2020, with the National Coronavirus Command Council enforcing a nationwide hard lockdown, initially meant to only be for 21 days (Ramaphosa 2020).

While the COVID-19 lockdown impacted and challenged many vulnerable communities, people experiencing homelessness were particularly vulnerable because of the lack of healthcare and social service resources (Geldenhuys 2021; Salisbury-Afshar et al. 2020). At the same time, the lockdown increased the visibility of homelessness (Mattsson et al. 2023). Once the hard lockdown was announced, those inhabiting the streets were required to find a place to stay. The directives to stay at home and practice virus mitigation measures were unrealistic and meaningless for people experiencing homelessness (Salisbury-Afshar et al. 2020). As a result, in Tshwane, around 2000 street-based homeless persons were rounded up by the police and placed at a stadium in the city centre. Initially, the living circumstances were dire, as there were insufficient ablution facilities, not enough blankets or food, poor adherence to social distancing and overcrowding. Heavy rains flooded tents, wiping out belongings and exacerbating the sanitation challenges (Geldenhuys 2021).

In response to this crisis, many stakeholders in the homeless sector became involved and collaborated to find solutions. This included CoTMM officials, the Tshwane Homeless Forum (THF), the University of Pretoria (UP), the University of South Africa, the Community Oriented Substance Use Programme (COSUP), various non-government organisations (NGOs) and faith-based organisations (FBOs), as well as churches and concerned citizens. Through the collaboration, street homeless people were placed at various shelters, including temporary shelters at tented parks, town halls and church halls, where they were able to receive shelter and food (Marcus et al. 2020; Renkin 2020). Of the almost 2000 people initially housed in the Caledonian Stadium, a large group consisted of opioid-dependent homeless people as well as those presenting with mental health symptoms (Stonehouse et al. 2023). While the initial emergency response focused on meeting immediate basic needs and medical needs (such as health screenings and substance withdrawal management), the lockdown provided an opportunity for people experiencing homelessness to access other services, including university-led occupational therapy interventions, which were otherwise not being provided in inner city homeless contexts in Tshwane, thereby triggering a focus on this underserved population.

In Tshwane, during Level 5 Lockdown (which involved drastic measures to contain the spread of the virus), occupational therapy interventions were initiated by staff from the UP in response to the unique needs arising from the temporary shelter settings. Given the limitations of lockdown level 5, these interventions included the development of booklets guiding the facilitation of life skills and leisure activities (developed by volunteer community service occupational therapists) and the provision of ‘doodles’ (various appropriate activities that could be accessed online, developed by an occupational therapist working in the homeless sector in Cape Town, specifically for lockdown) to facilitate meaningful engagement of the shelter residents. Activity toolkits were also developed to facilitate group therapy and constructive use of time for residents in homeless settings, with three online training webinars run for shelter staff (especially social workers [SWs] and social auxiliary workers) who were working on the frontline during the lockdown, so that the occupational therapy-supported interventions could be facilitated by them at the various shelters during lockdown levels 5 and 4.

Over the following weeks, the various changes to lower lockdown levels allowed for increased service delivery. At level 3 lockdown (which still retained restrictions on many activities), final-year occupational therapy students from the UP were permitted by the institution to restart their work-integrated learning (WIL), where they provide occupational therapy services under the supervision of community-based occupational therapists and academics as part of their exit-level requirements. Some of these students were placed in various inner-city settings in August 2020 in response to the ongoing need for occupational therapy services. Student-led occupational therapy interventions were conducted at homeless settings such as temporary shelters, transitional housing facilities and drop-in centres, as well as inner city COSUP sites (a community-based substance-use harm reduction project, funded by the CoTMM and implemented by the UP Community Oriented Primary Care Research Unit). Occupational therapy intervention by students included mostly activity-based socio-emotional group therapy addressing aspects pertaining to social skills (such as communication skills, conflict management, teamwork, building trust), life skills (e.g. interpersonal skills, managing thoughts, financial management, goal setting) and pre-vocational skills (such as time management, work habits, problem solving), presented in response to expressed needs. These interventions continued at selected homeless settings for lockdown levels 2 and 1 (where most normal activity was allowed to resume with precautions and health guidelines in place), and beyond, with students continuing to provide intervention in many of the remaining homeless settings to date.

The contribution of university-led occupational therapy interventions in homeless settings in Tshwane needed to be explored to understand the benefits and challenges experienced, as well as to establish strategies for future planning and identify what occupational therapy interventions in homeless settings in Tshwane should entail.

### Aim

The aim of this research was to explore the experiences and perceptions of stakeholders (people experiencing homelessness, homeless sector practitioners and occupational therapy students) as well as barriers and challenges and benefits and facilitators of university-led occupational therapy interventions in homeless settings (such as shelters, drop-in centres and transitional housing projects) in Tshwane during COVID-19 lockdown and beyond, up until March 2023.

### Rationale

The contribution of occupational therapy in homeless settings in Tshwane began within a disaster-management context, but has subsequently evolved. To inform future practice, there was a need to better understand the contribution of university-driven occupational therapy in these settings. In particular, the experiences and resulting perceptions of people experiencing homelessness who participated in student-led occupational therapy intervention and other key stakeholders in Tshwane needed to be explored. Little is known about the challenges, barriers, benefits and facilitators of occupational therapy interventions in South African homeless settings from both recipients and service provider perspectives. Understanding stakeholders’ experiences and resulting perceptions of occupational therapy intervention within homeless contexts in Tshwane will enable occupational therapists to better meet the needs of people experiencing homelessness.

### Theoretical framework

This study used the Capability Approach (Wells & Wrenn n.d.) as a theoretical framework to guide an understanding of the value of occupational therapy interventions for people experiencing homelessness (Garza-Vazquez & Deneulin 2019). This person-centred approach consists of the means to achieve (Inputs and/or Characteristics), the freedom to achieve (Capabilities) and the achievement (Functionings) (Munger, Macleod & Loomis 2016). An individual’s freedom to achieve well-being is related to what people can do and be, the opportunity and ability to achieve, and the freedom to choose the ‘functionings’ one values, which overlaps with the concerns, values and key features of occupational therapy. Thus, the approach highlights how participants convert their actual ‘doings’ and ‘beings’ with the inputs, characteristics or opportunities available into real freedoms or capabilities, that is, what the participants are actually able to do and be through the opportunities available to them, which, in this study, includes the access to and participation in occupational therapy intervention (Alkire n.d.; Garza-Vazquez & Deneulin 2019).

## Research methods and design

### Study design

An explorative, descriptive qualitative research design (Creswell et al. 2007) was used to understand stakeholders’ experiences and perceptions regarding the benefits and facilitators of, and challenges and barriers to, occupational therapy interventions in homeless settings between April 2020 and March 2023.

### Population

The population consisted of stakeholders that included people experiencing homelessness who were staying in homeless settings (such as temporary or permanent shelters, transitional housing facilities, drop-in centres) at the time of the study, homeless sector practitioners (such as shelter managers [SMs], SWs, volunteers), and final-year occupational therapy students from the UP conducting their community-based WIL in these settings.

Only people experiencing homelessness who resided in homeless settings (such as temporary shelters) or who made use of services at homeless settings (such as drop-in centres) where university-led occupational therapy services were provided were considered for this study.

### Study sample and sampling method

Both convenience and purposive sampling were used to identify participants. Purposive sampling was used to identify homeless settings based on existing working relationships with homeless practitioners such as staff from COSUP sites and NGO/FBO-run settings. Owing to the chaotic manner in which some temporary homeless shelters were established in the early stages of the COVID-19 lockdown, together with the high turnover of people experiencing homelessness accommodated in these shelters, with some of these shelters closing as lockdown levels lifted, convenience sampling was necessary to recruit participants. Thus, stakeholders working at- or residing in homeless settings, falling mostly within Region 3 (which includes the centre or inner city of the metropole where many homeless settings are located) in Tshwane, who had experience of occupational therapy intervention, and who were willing to participate and capable of giving informed consent were asked to be part of the research.

Occupational therapy intervention included the initial supported intervention during lockdown levels 5 and 4, in which the first author (S.A.) participated as a volunteer, and the second author (H.E.L.) had facilitated the training of practitioners. Intervention also included student group-based intervention through WIL, where, from August 2020 to March 2023, 54 students have each worked for 6–7 weeks in these settings. The students’ WIL supervisor was the third author (M.N.S.J.v.R.), who had built a working relationship with homeless practitioners through the homeless network she was involved in, and was, thus, able to place occupational therapy students at these settings.

Purposive sampling was also used for documentation (student analysis, intervention planning, session evaluation, handover documentation, as well as feedback forms completed by participants), which was part of WIL requirements for student-led occupational therapy interventions from level 3 lockdown.

### Study setting

The study was conducted at selected homeless settings within the CoTMM, where occupational therapy interventions occurred during the COVID-19 lockdown levels and continued after restrictions were lifted to the time of writing this article. Sites were selected from each level of lockdown to ensure a representation of the various types of shelters and occupational therapy interventions.

Occupational therapy-supported intervention:

- Levels 5 and 4: Temporary shelters included NGO- and FBO-run facilities, churches, town halls and tented sites. Occupational therapy was not classified as an essential service at this stage; therefore, no formal services were provided. This led to the initiation of volunteer occupational therapy-supported interventions by therapists aligned with UP.

Student-led occupational therapy group intervention, conducted by 4th-year occupational therapy students under supervision as part of WIL:

- Level 3: Once community-based WIL resumed, occupational therapy students began working at homeless settings throughout the city. Students ran occupational therapy group sessions at several shelters, drop-in centres, and supported housing for destitute older persons that had been established during lockdown. Several temporary shelters closed as the lockdown limitations were lifted.
- Levels 2 and 1: The sites that continued to function were shelters and drop-in centres run by NGOs/FBOs. Occupational therapy group intervention run by students continues to take place in many of these settings to date.

Table 1 shows the homeless settings where university-led occupational therapy services were implemented in Tshwane from lockdown and during the data collection period, with seven specific sites selected for the study.

**TABLE 1:** Homeless settings and occupational therapy intervention provided.

Homeless settings	Lockdown level	Intervention	Data obtained and data collection methods	Specific site(s) selected
Temporary and transitional shelters, including those run by NGOs and FBOs, situated in churches, town halls and tented settings	5 and 4	Occupational therapy-supported intervention: Doodles Activity packs and toolkits Online training	Feedback through notes and transcriptions from various stakeholders obtained as part of monitoring during implementation (four recorded interviews and one focus group discussion with four people experiencing homelessness)	One site selected: A temporary shelter in church hall that closed after level 4
	3	Occupational therapy student-led group therapy sessions	Student analysis, intervention planning, session evaluation, handover documentation; group session feedback forms, compiled during WIL	Two sites where occupational therapy students worked during level 3, but did not continue to work there after level 3: (1) a temporary shelter in a town hall; (2) a newly established FBO-run transitional shelter for older or disabled men
	2 and 1	Occupational therapy student-led group therapy sessions	Student analysis, intervention planning, session evaluation, handover documentation; group session feedback forms, compiled during WIL	Three sites were selected where occupational therapy students worked during levels 3, 2 and 1: (1) a shelter for women survivors of gender-based violence (and their children); (2) a shelter for men who use substances; (3) a shelter for destitute mental healthcare users
	Sites where occupational therapy intervention was conducted beyond lockdown	Occupational therapy student-led group therapy sessions	12 interviews with key stakeholders where occupational therapy intervention continued till March 2023 Two focus group discussions with people experiencing homelessness (five and three shelter residents, respectively) Student analysis, intervention planning, session evaluation and handover documentation; group session feedback forms, compiled during WIL	Three sites were selected where students were working at the time of the study. The same two shelters mentioned in the previous row i.e. (1) and (2), as well as a shelter for destitute older persons (men and women)

FBO, faith-based organisation; NGO, non-government organisation; WIL, work-integrated learning.

### Data collection tools

Three qualitative data collection tools were used during the study: (1) semi-structured interviews, (2) focus group (FG) discussions and (3) document analysis. Together, these methods enabled the study to address its research aim.

The interviews held with homeless practitioners focused on their experiences of and perceptions on the occupational therapy interventions offered at their respective sites. The interview guide consisted of four sections: (1) experiences and perceptions of occupational therapy interventions, (2) benefits and facilitators, (3) the perceived influence of the interventions and (4) challenges and barriers encountered.

Focus groups with individuals experiencing homelessness sought to understand the influence of occupational therapy intervention in participants’ lives. The semi-structured guide for these discussions covered (1) experiences and perceptions of occupational therapy intervention, (2) influence of the interventions and (3) challenges relating to the interventions.

Document analysis drew on documentation produced by 4th-year occupational therapy students during their WIL community placements. These documents included the setting analysis, intervention plans, session evaluations, reflections and handover documentation compiled while students facilitated group sessions in homeless settings during lockdown levels 3, 2 and 1 of lockdown (August 2020 to April 2022) as well as the period that followed, up to March 2023. Stakeholder feedback forms that were completed by homeless practitioners that the students worked with and group therapy members to evaluate student-led group interventions per WIL block were also analysed. Additional documentation, such as general site information, feedback reports, community projects and presentation slides, was also included, where applicable.

### Data collection

Data were collected by the first author (S.A.), an occupational therapist conducting research for her master’s degree. The second author (H.E.L.) connected the researcher to relevant early frontline practitioners, and the third author (M.N.S.J.v.R.) introduced the first author to relevant sites and practitioners. Across these settings, the first author conducted 16 semi-structured interviews, three FG discussions, and relevant document analysis. Data collection and analysis occurred concurrently to allow for ongoing assessment, with saturation reached once no new codes or themes emerged from the final interviews and FGs (Saunders et al. 2018).

Data collection entailed two parts:

Part 1: Interviews and FG discussions

1.1. Four previously recorded interviews and one FG discussion with stakeholders, originally undertaken during lockdown alert levels 5 and 4 as part of monitoring and evaluation activities, were included. The transcripts of these recordings were reviewed for relevance to the current study and incorporated into the dataset.

1.2. Twelve interviews and three FGs were conducted with key stakeholders who had facilitated intervention (homeless practitioners) or had participated in occupational therapy interventions (shelter residents and drop-in centre attendees) during lockdown levels 3, 2 and 1 up until March 2023. These were conducted on separate days and across various sites to allow for broad participation and to support reaching data saturation. All sessions were conducted in English with translation support provided by a postgraduate student at one FG discussion where some vernacular words were used. Discussions were audio-recorded and transcribed verbatim by a research assistant.

Part 2: Document Analysis – 88 sets of written documentation compiled by occupational therapy students during their WIL placements at inner-city sites were accessed and analysed. This documentation included intervention plans, session evaluations, reflections and feedback forms (62 documents), and 26 handover reports.

### Data analysis

The process of thematic analysis commenced with the receipt of each data set. Reflexive thematic analysis was used as the overarching approach for coding and theme development, emphasising researcher flexibility and reflexivity (Braun & Clarke 2006). This included the steps of familiarisation with the data through active and repeated reading of transcripts and documentation; generating initial codes; searching for themes by analysing, combining and comparing codes; and reviewing, refining and defining themes.

While generating themes, the researcher moved iteratively between data and theory (the Capability Approach) to explain findings and deepen interpretation, thus using abductive thematic analysis (Thompson 2022). This process was done using ATLAS.ti and included the transcriptions of all interviews and FG discussions as well as the documentation.

While the researcher made brief reflective notes after each data collection session to document key impressions and contextual details, no formal observational notes were made or data systematically collected. These reflections were used primarily to support reflexivity and interpretation during data analysis.

### Trustworthiness

The researcher (first author) spent significant time in the settings and focused in detail on the most relevant elements through persistent observation. She was able to corroborate her findings from the FGs, interviews and document analysis with her research supervisors, who knew the settings well. Multiple data sources (various stakeholders and occupational therapy intervention planning documentation) and methods of data collection (interviews, FGs and document analysis) provided an opportunity for triangulation and ensured rigour.

Initial gatekeeper permission was obtained from the managers of the respective shelters, and written informed consent was obtained on an individual basis for each participant, per interview or FG discussion after participants were informed about the purpose and process of the study. All participant details were deidentified by using coded identifiers. Access to students’ written documentation (already deidentified) was granted by the WIL supervisor. All data were securely stored on a password-protected laptop.

## Results

### Demographic information

Table 2 outlines the participant groups, which included people experiencing homelessness, homeless practitioners and occupational therapy students. All participants took part in either interviews or FG discussions. To protect confidentiality, abbreviated pseudonyms were used, and quotations are attributed to stakeholders from individual interviews, the FGs or extracts from occupational therapy student documentation.

**TABLE 2:** Stakeholder pseudonyms and settings.

Stakeholder	Pseudonym and abbreviation	Setting	Participation
Shelter Manager/Pastor, Male	SM/P-G	Temporary shelter	Interview 1
Pastor, Female	P-J		Interview 2
Site Manager M, Male	SM-M		Interview 3
Site Manager T, Male	SM-T		Interview 4
Participant 1 Participant 2 Participant 3 Participant 4	P1 P2 P3 P4	Men’s shelter (including PWUD)	Focus Group 1
Occupational Therapy Students	OT-S	Homeless Settings from lockdown level 3, 2, 1	Document Analysis
Social Worker L, Female	SW-L	Transitional housing for MHCU	Interview 5
Social Worker T, Female	SW-T	Long-term housing for destitute elderly	Interview 6
Homeless individual K, Male	HI-K	Transitional housing for MHCU	Interview 7
Area Manager E, Male	AM-E	Men’s shelter (including PWUD) and work site for shelter residents	Interview 8
Community Health Worker C, Female	CHW-C	Shelter (including PWUD)	Interview 9
Site Manager I, Male	SM-I	Work site for shelter residents	Interview 10
Site Officer P, Male	SO-P	Work site for shelter residents	Interview 11
Homeless Individual N, Male	HI-N	Shelter (including PWUD)	Interview 12
Nurse T, Male	N-T	Long-term housing for destitute elderly	Interview 13
Social Worker M, Female	SW-M	Transitional shelter for women	Interview 14
Auxiliary Social Worker L, Female	ASW-L	Transitional shelter for women	Interview 15
Social Worker P, Female	SW-P	Long-term housing for destitute elderly	Interview 16
Participant A Participant B Participant C Participant D Participant E	PA PB PC PD PE	Transitional housing for MHCU	Focus Group 2
Participant X Participant Y Participant Z	PX PY PZ	Long-term housing for destitute elderly	Focus Group 3

PWUD, people who use drugs; MHCU, mental health care users.

### Themes and sub-themes

The study findings present the benefits and facilitators of, and challenges and barriers to, occupational therapy intervention with people experiencing homelessness in homeless settings, based on the experiences and perceptions of stakeholders, and framed within the Capability Approach. Table 3 shows the themes generated through the reflexive thematic analysis.

**TABLE 3:** Themes and sub-themes generated through reflexive analysis.

Themes	Sub-themes
1. Perceptions of stakeholders regarding occupational therapy intervention	1.1: The intervention itself Types of group therapy sessions Group therapy topics The power of activities Occupational therapy student as outsider 1.2: Significance of occupational therapy groups Need for occupational therapy groups (filling the gaps) Value of occupational therapy groups
2. Benefits and facilitators of occupational therapy intervention	2.1: A springboard The interprofessional team Creating structure and routine Compulsory group attendance Meeting needs 2.2: Through the lens of occupational therapy Philosophy of client-centred care Principles of occupational therapy Therapeutic use of self Therapeutic use of activity Building a therapeutic relationship 2.3: Influence of occupational therapy groups Skills development and carry over Development of coping mechanisms Improved independence Facilitation of curative factors Changes in motivation and attitude Community reintegration
3. Challenges and barriers to occupational therapy intervention	3.1: States of beings and doings Unique needs of PEH Challenges PEH face Lack of volition Difficulty establishing rapport 3.2: Demographics Language barriers Cultural competence 3.3: Logistics Group attendance Group participation Group size and duration Limited resources Lack of continuity of intervention

PEH, people experiencing homelessness.

**FIGURE 1:**
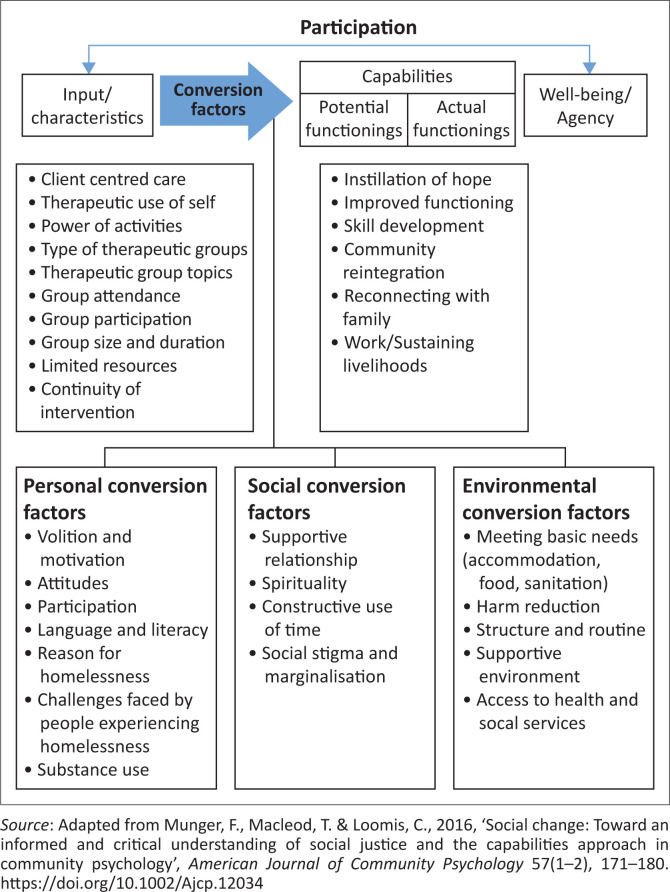
Occupational therapy interventions in homeless settings framed within the Capability Approach.

The themes and sub-themes were then aligned to the Capability Approach through abductive analysis in terms of: Input/characteristics, Conversion factors (personal, social, and/or environmental), and Capabilities (potential and actual functionings) that could lead to well-being and agency for people experiencing homelessness (see Figure 1).

### Inputs and/or characteristics

The positive inputs and/or characteristics that facilitated productive and friendly interaction related to the philosophical stance taken by the occupational therapy students and their WIL supervisors to be client-centred, approachable, non-judgemental and open to change, as informed by the needs of people experiencing homelessness. One of the cornerstones of occupational therapy is the therapeutic use of self, and through building rapport, even and especially as an ‘outsider’, occupational therapy students and supervisors were able to provide opportunities for meaningful engagement. Participants expressed the need for a ‘specific type of OT student’, in that they should ‘not be too pushy, demanding or questioning’, but continue to allow people experiencing homelessness ‘to be free and express themselves as they deemed fit’ and, thus, occupational therapy students were seen to play a ‘major role…in the group, in people’s lives’ (SM-I, male, Interview 10). The participants appreciated the non-judgemental attitudes of the occupational therapy students towards people experiencing homelessness.

At times, language barriers were challenging, as well as occupational therapy students not understanding homelessness. Additionally, being young was a disadvantage as,

‘…there were always those dynamics to say, I’m from prison there is nothing you can tell me, I know better. And some will say I’ve lived in the streets for so long you cannot tell me nothing [*sic*], you don’t know nothing about life, you have never lived on the streets.’ (SM-M, male, Interview 3)

Through immersion in the homeless settings and the development of therapeutic relationships, the students were able to develop insight regarding the complexity of homelessness.

Another cornerstone of occupational therapy intervention is the therapeutic use of activities for meaningful engagement and participation, and the ‘power of activities’ to facilitate therapeutic outcomes, as opposed to only discussion groups. Activity-based groups that involved a task or social element and groups incorporating skill building, which is end-product focused, included various topics relating to life skills and the constructive use of time. For example, a homeless practitioner working with people who use drugs (community health worker [CHW-C], female, Interview 9), confirmed, ‘they don’t want to sit and just listen most of the time. They want to listen and do something…’. Meaningful activities were noted to be enjoyable, thus lift participants’ energy, and elevate their mood. Activities were also noted to create togetherness. Participant A (male), a resident from a shelter for mental healthcare users, confirmed during FG2 the ‘power of activity’ through its ability to create ‘togetherness … [*as it*] bring[*s*] everyone closer together. Even if it’s just for that little while’.

The topics of group sessions were in response to needs expressed by group participants. Topics were relevant and meaningful and were categorised into group members’ ‘occupational performance areas’ – in other words, personal management, social ability, work ability, and the constructive use of time. A needs assessment ensured that topics were relevant – ‘every group [*of students*] comes with different ideas’, and ‘what they’ve been teaching …, it’s always been something new’ (CHW-C, female, Interview 9). A group member agreed, ‘… [*the group sessions*] were very different. Very, very different. And strangely enough, they never ever repeated somebody else’s work. I don’t know how you work that out’ (Participant Y, male, resident at facility for destitute elderly, FG3).

The value occupational therapy groups brought to homeless settings, particularly the homeless practitioners, was seen through positive expression about the groups: ‘I love the groups’ (SW-L, female, Interview 5). The group sessions were noted to be unique, and were perceived as beneficial and valuable through the ‘process of engagement’ (SM-I, male, Interview 10). The opportunity for group members to express themselves through activities was important, and healing was facilitated through the occupational therapy groups: ‘…because some people, they underwent difficult circumstances whereby they’re hurt deep inside. So, they require some sort of healing. So, they’re bringing that healing process within the group…’ (SM-I, male, Interview 10). Occupational therapy groups ‘won them over’ (Nurse N-T, male, Interview 13), and helped to overcome group members’ despondency and trust issues. Homeless practitioners benefitted from the occupational therapy groups, ‘A lot, a lot. I don’t see how we would have survived without the OT [*occupational therapy*] students…’ (Nurse N-T, male, Interview 13). The value of the intervention was further highlighted through the gratitude and appreciation expressed by group members towards the occupational therapy students regarding their presence and willingness to work with shelter residents.

Group attendance and participation were challenges when group attendance was erratic and unpredictable, but were facilitators when group members attended regularly and engaged within the group. Group attendance and participation fluctuated over time, and participation was influenced by residents’ attitudes (eager versus uninterested) and motivation (adequate versus poor); presence or absence of physical disabilities (those using wheelchairs and navigating an outdoor and uneven terrain), and rapport or level of trust built between the students and the group participants. Compulsory group attendance, which was implemented in some settings, not only facilitated group attendance, but also participation and exposure to potential growth opportunities for shelter residents.

Other challenges and barriers to occupational therapy intervention included the logistics of occupational therapy groups, such as problem group members, disrupted group sessions, fluctuating size of the groups, and varying group attendance, as well as limited resources and occupational therapy services not being continuous because of the nature of WIL scheduling and the lack of permanent occupational therapists. ‘We are always sad because we know they’re here short term, and it’s always that’ (SW-L, female, Interview 5).

### Conversion factors

Personal conversion factors related to group members’ attitudes and volition acted either as barriers or facilitators to their engagement in occupational therapy intervention. These attitudes directly shaped participants’ levels of involvement in the groups. One homeless practitioner described how both positive and negative attitudes were present among group members, noting that these attitudes affected one another and often shifted over time as individuals continued to attend the occupational therapy groups. People experiencing homelessness have pertinent challenges, and the reasons for their homelessness often differ significantly. As a result, their needs are highly individual. For example, a person experiencing homelessness because of problematic substance use may have very different priorities and support needs compared to someone who became homeless after losing their job and is unable to afford rent.

Social conversion factors include the supportive relationships that emerged among group participants through group therapy, enhancing the development of coping mechanisms and improving mood and motivation.

‘They seem to help us make connections. With one another … But you know I … you felt good, and you’re a little but more forthcoming, you know. The person [*other homeless persons*] is not quite the enemy that he or she was.’ (PY, male, FG3)

Furthermore, life skills groups were perceived as beneficial and facilitated positive changes, ‘A response to the information they were receiving from OT [*occupational therapy*] students, that there was a shift, a change in behaviour’ (SW-L, female, Interview 5). PE, male, FG2, confirmed this, ‘What they forget is that they’re helping change my attitude…towards other people’. ‘There was [*sic*] other people, especially when I don’t feel well, then I will just ignore them or whatever. But they [*occupational therapy students*] helped me keep calm…’.

Several of the settings were run by FBOs, and the inherent grounding in spirituality in those spaces also enhanced and served as a facilitator to occupational therapy intervention, as it not only appeared to make people experiencing homelessness more receptive of interventions, but also gave group members a sense of acceptance, self-worth and, ultimately, hope through the application of curative factors such as universality, imparting information and cohesion.

‘Even if you want to take religion out you have to put in what the spirituality side was, which was affirming. Making the person understand the importance of them been seen to be human.’ (Temporary SM/P-G, male, Interview 1)

The sense of acceptance, community, non-judgement and hope translated through the occupational therapy group sessions reinforced the supportive environment of the shelters.

In terms of environmental conversion factors, the provision of structure and routine within the homeless setting and a supportive environment are important: ‘It’s very helpful for me. It keeps me going, and it keeps me motivated. It give[*s*] me the reason the wake up every morning’ (Participant D, male, FG2). The provision of basic needs through accommodation, food and hygiene facilities, health and psychosocial care, as well as assisting with important documentation and helping to build relationships among people experiencing homelessness, combined with the occupational therapy intervention itself, enabled people experiencing homelessness with potential freedoms or opportunities. Being able to access other health services was valuable to residents.

‘…there was another one who was using one hand, … the left hand was working, the right hand [*was*] burn[*t*] … in the beginning, he didn’t want to, he was not encouraged, like, he was not even motivated to use his hands… But then after that … I said to them [*occupational therapy students*], “guys please just talk to this guy because he doesn’t seem motivated.” They brought the skills, life skills and now he was able to wash his own clothes … to clean the room on his own.’ (ASW-L, female, Interview 15)

Interprofessional teams and a harm reduction paradigm also facilitated and enhanced occupational therapy intervention in that it created a ‘very balanced kind of input – with the input working on a number of different levels’ (SM-G, male, Interview 1). People experiencing homelessness who used opioid drugs were in a better state of ‘being’ because of the physiological benefits of opioid substitution therapy, thus promoting participation in occupational therapy intervention as well as the potential to benefit from the groups.

### Capabilities

When inputs, characteristics or resources and conversion factors were in place, such as adopting a client-centred approach, using therapeutic activities, ensuring positive volition of homeless individuals, and a supportive environment, it not only positively influenced outcomes but also enhanced the capabilities of people experiencing homelessness. Conversely, an unsupportive environment, language and literacy barriers, unresponsive attitudes and poor motivation, and lack of continuity of occupational therapy intervention negatively affected outcomes and were challenges/barriers to occupational therapy intervention.

Improved independence was noted because of occupational therapy groups, with ‘a lot of changes seen in terms of exit [*from the shelter*]’ (ASW-L, female, Interview 15).

‘When they first came here, when they attended classes [*group therapy*], and before, there is a huge difference. Even physically, with the way they dress, when they first come here, they are hopeless and all that, now you will see them starting to have that interest of looking nice, going for a job search [*sic*] and, it’s all because of the impact of the [*occupational therapy*] student[*s*] and social worker.’ (SW-M, female, Interview 14)

In terms of reintegration,

‘Because in terms of employment, the OTs [*occupational therapy students*] are still focusing on helping them to get back into [*the*] work environment. I can say for them to go home, it shows the positive influence of the occupational therapy and other activities, because … it was not just an easy decision to make, to go home.’ (SM-T, male, Interview 4)

Reintegration into the community, reuniting with family, seeking employment/work or maintaining sobriety ultimately resulted in improved functioning and well-being (see Figure 1).

Given the value and perceived benefit of occupational therapy groups, a need for occupational therapy intervention in homeless settings on a more permanent, long-term basis was identified. ‘It’s needed actually. If it was possible we would have one OT [*occupational therapist*] here every single day for our clients’ (CHW-C, female, Interview 9). One stakeholder identified the need for individual, one-on-one sessions, especially because digging deeper into one’s life ‘cannot be explained in public’ (SM-I, male, Interview 10). Even if not a permanent occupational therapist, the need for more students at the placements, as well as more regular sessions, was identified by shelter residents in written feedback forms.

## Discussion

The findings identified various personal, social and environmental experiences that contributed to improved functioning, as well as benefits and facilitators, and challenges and barriers in implementing university-led occupational therapy intervention among people experiencing homelessness in homeless settings in Tshwane.

People experiencing homelessness are often excluded and prevented from engaging in the things that bring meaning to them (i.e. their ‘occupations’) in the community, with their energy directed towards meeting their basic needs, such as food and shelter and surviving on the streets. This results in ‘occupational deprivation’ and alienation, which may contribute to a loss of meaning to life (Eakman 2015; Lloyd & Bassett 2012). In the belief that ‘doing brings meaning’, occupational therapists aim to enhance engagement in meaningful occupations (those things that people need to, want to, and/or are expected to do), which allows people experiencing homelessness to address occupational needs (such as sustaining livelihoods, the constructive use of time, and accessing health and social services), improving their physical and mental health, contributing to improved overall functioning and wellbeing (Heuchemer & Jospehsson 2006; Lloyd & Bassett 2012). Wilcock (1999) seminally states that occupational therapists focus on the therapeutic use of meaningful occupation to promote health, well-being and participation in life, and in so doing, assist individuals to transform their lives through a synthesis of ‘doing, being, and becoming’. The occupational therapist’s expertise in activity analysis, environmental modifications and compensatory strategies provides a unique perspective, contributing to an individual’s functioning and optimal independence (Lloyd & Bassett 2012).

Occupational therapists adopt a holistic and person-centred approach, with their role-specific skills well-matched to address the basic and occupational needs of people experiencing homelessness (Grandisson et al. 2009). Occupational therapists address the performance of activities of daily living (e.g. self-care, hygiene), instrumental activities of daily living (e.g. money management, community mobility), social participation, work, leisure and education (Barnekow & Pickens 2011), and they assist to meet the occupational needs of people experiencing homelessness, including survival, identity, social connectedness, self-care, and rest and sleep. Apart from the emerging roles of outreach worker, advocate and case manager, the intervention provided by the occupational therapist includes practical, occupational and family support, individual sessions and support, group work and specific skills training, aiding the development of motivation (Chard, Faulkner & Chugg 2009; Helfrich & Fogg 2007; Lloyd & Bassett 2012; Muñoz, Dix & Rechenbach 2006a; Muñoz et al. 2006b; Parmenter 2003; Parmenter, Fieldhouse & Barham 2013). It is the occupational therapist’s holistic skillset with specialised knowledge of functioning in relation to health conditions, populations and the impact of the environment that enables people experiencing homelessness to be empowered to participate in their meaningful roles and routines (Van Oss et al. 2020). The inclusion of occupational therapy services has been found to bring purpose and incorporate routine and wellbeing to marginalised populations, such as in homeless contexts (Synovec 2021).

In this study, through occupational therapy group intervention, people experiencing homelessness staying in homeless settings were provided with the opportunity to practice the skills (such as building and establishing trust, conflict management and goal setting) needed to gain control over their lives, while enabling decision-making, improving motivation and fostering hope. Occupational therapy intervention served as a resource for participants through inputs that were person-centred, by using therapeutic activities, and having a good understanding of how occupational deprivation and injustice can impact people experiencing homelessness. What participants did with – or how they responded to or participated in – the intervention was impacted by the interaction of the respective conversion factors in place, such as a supportive environment and ensuring positive volition, which enhanced participants’ sphere of capabilities, resulting in ‘functionings’ to facilitate improved well-being.

Therefore, a significant match is identified between the occupational needs of homeless individuals and the specific skills and values of occupational therapists, and a clear and relevant role can be seen for occupational therapy services with the homeless population (Van Oss et al. 2020). While, historically, occupational therapy has not been significantly involved with supportive housing interventions in South Africa, the profession could be a valuable asset at all phases of homelessness and when maintaining an individual’s transition to housing because of the ability to address life skills and facilitate achieving optimal functioning (Raphael-Greenfield & Gutman 2015).

Integrating occupational therapy interventions into these spaces, as in this study, reduces barriers and increases access to care and services, as the therapists work where the people experiencing homelessness live, making it possible for them to access their services. This puts therapists in an ideal position to build knowledge, awareness and understanding of the context of people experiencing homelessness and how it affects their occupational engagement, enabling therapists to meet people where they are at and address their needs by providing best practices. Furthermore, with the rise in homelessness, many people are experiencing – or are at risk of – physical and/or mental health issues; it is appropriate that occupational therapists consider how to meet the needs of this diverse but marginalised population.

The types of homeless settings also shaped the needs of people experiencing homelessness and, thus, the nature of interventions required. While many programmes have included professions such as SWs, nurses, peer supporters and housing specialists, one valuable profession is often overlooked. Occupational therapy assists people to develop or re-establish the complex skills they need, and can motivate people experiencing homelessness and instil hope that, by actively taking steps aligned with their needs and capabilities, they can rebuild their lives. Living without a home affects not only other people’s perceptions, but also how individuals view themselves, influencing multiple aspects of their functioning. The purpose of occupational therapy is to improve people’s occupational engagement and participation in meaningful activities, ultimately enabling people to find meaning, purpose, and live to their own determined potential in a way that upholds their dignity.

The collaboration demonstrated by the UP, THF and the CoTMM, together with various NGOs, FBOs and other stakeholders in various homeless settings, led to several gains. This collaboration, which included occupational therapy, built a strong capacity to achieve largely positive outcomes in addressing homelessness in the inner city of Tshwane. Through shelter placement, the provision of health and psychosocial services that included occupational therapy interventions, street-based persons were able to access support that had previously been illusive, and, while not everyone made use of the opportunities, the availability of services needs to be ongoing to enable people to access these when the time is right.

### Study limitations

- Because of the numerous challenges experienced by shelter staff during these initial lockdown levels, it remains uncertain how much of the supported occupational therapy intervention was delivered at the various sites during levels 5 and 4.
- Interviews regarding the occupational therapy-supported intervention from lockdown levels 5 and 4 were conducted several months later, which could have resulted in recall bias.
- Some of the homeless participants who started the intervention did not complete it, which means that the opinions of people who may not have found it useful will not have been recorded.
- Residents in homeless settings were mostly not obliged to participate in the occupational therapy group sessions, which means that only those who attended gave their opinions.
- Because of the transient nature of the settings, there was limited demographic information of group therapy participants, and thus generalisations cannot be made.
- The study sample was, to some extent, influenced by the researcher’s availability because of various delays in the project as well as time constraints when visiting the homeless settings and the availability of stakeholders at that time.
- Interviews and FG discussions were conducted in English. While this was not a barrier regarding the homeless practitioners, it may have been a barrier for some FG participants. At all FGs, there were group members present who could have assisted with basic translations, if necessary.

### Recommendations

Regarding occupational therapy practice and policy improvement, occupational therapy should be listed as an essential service, especially in situations when meaningful engagement or functioning is disrupted, such as in a pandemic, so that people experiencing homelessness are able to access services. Through engagement in occupation (that which brings meaning to one’s life), people experiencing homelessness can cope with their changed circumstances and achieve improved health, wellbeing and participation in life.

Shelters should become resource centres for people experiencing homelessness to access health and support services that include occupational therapy as part of a holistic approach to care. The initial opportunity to implement occupational therapy services in homeless settings in Tshwane as a result of the COVID-19 lockdown needs to be built on further, as it contributes to improving meaning and purpose through meeting people’s functioning needs. At the time of writing this article, a specific non-profit organisation using an occupational therapy-led approach opened a centre in Tshwane, building on their work in Cape Town and Johannesburg. This research hopes to contribute to building occupational therapy services in the homeless sector and support further research.

Further research needs to be conducted regarding the role of occupational therapy in the different types of homeless settings in South Africa, which would contribute to the National Homeless Policy being developed to provide holistic services for the growing number of people experiencing homelessness in the country. Services and research need to be interdisciplinary so that interventions can be provided by a combination of disciplines, rather than in isolation, given that the needs of people experiencing homelessness vary over time and space.

In terms of education and continued professional development of occupational therapists, there are some clear training needs that emerged. These include understanding the unique realities of people experiencing homelessness and homeless contexts, as well as harm reduction as a strategy for people experiencing homelessness who use drugs. In undergraduate training, this can be best facilitated through immersion in these settings through WIL and academic service learning.

## Conclusion

There are thousands of people experiencing homelessness in South Africa. The struggling economy makes it difficult for many to find a way out of poverty. In the absence of adequate support, people experiencing homelessness are often left to their own devices to try to improve their lives, with some simply being unable to do so because of their circumstances or health challenges. The need for holistic health intervention that includes occupational therapy was identified in homeless settings in Tshwane during the lockdown, and this study demonstrated the benefits of university-driven occupational therapy services. Access to necessary health and social services, shelter and affordable social housing are key social determinants of health, so that people experiencing homelessness can function and participate meaningfully in life while navigating pathways out of homelessness.
